# Supplementary material for: The mechanisms of siRNA selection by plant Argonaute proteins triggering DNA methylation
Source: Nucleic Acids Res. 2022 Dec 7;50(22):12997–3010. doi: 10.1093/nar/gkac1135 (PMC9825178; doi:10.1093/nar/gkac1135)
Supplement: gkac1135_Supplemental_Files [file gkac1135_supplemental_files.zip › Supplementary information_revision_final.pdf]

Supplementary information

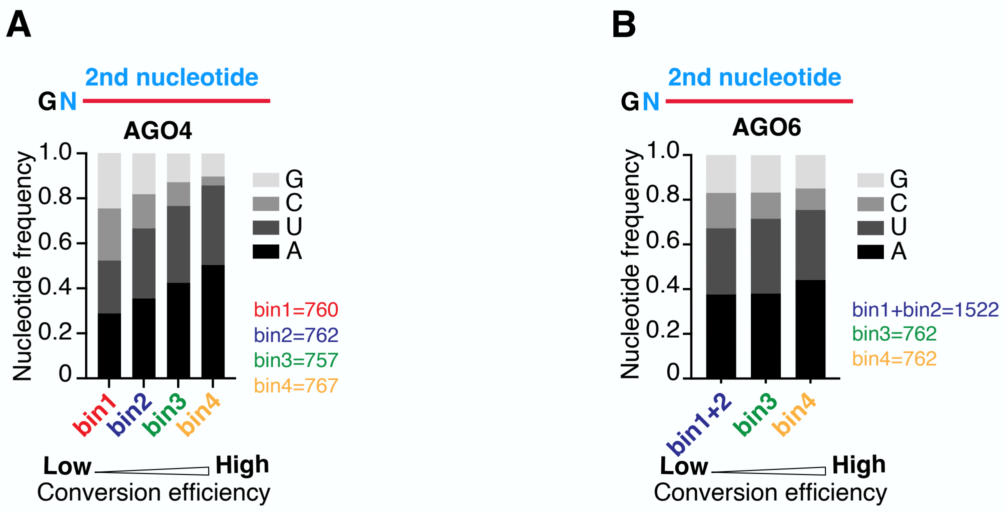

Supplementary Figure 1. Second nucleotide frequency of AGO4- and AGO6-bound 5′ G small RNAs.

Second nucleotide frequency of AGO4- (A) and AGO6- (B) bound 5′ G small RNAs. Number of P4RNAs in each bin is shown on the right side. Since those P4RNAs with 0 match to the AGO6-bound 5′ G small RNAs exceed the capacity of a single bin, we merged the bin 1 and bin 2 as one bin. P4RNAs with higher CEs tend to have an A/U nucleotide compared to a C/G nucleotide at the g2 position in both AGO4 and AGO6. See also Supplementary Table 3.

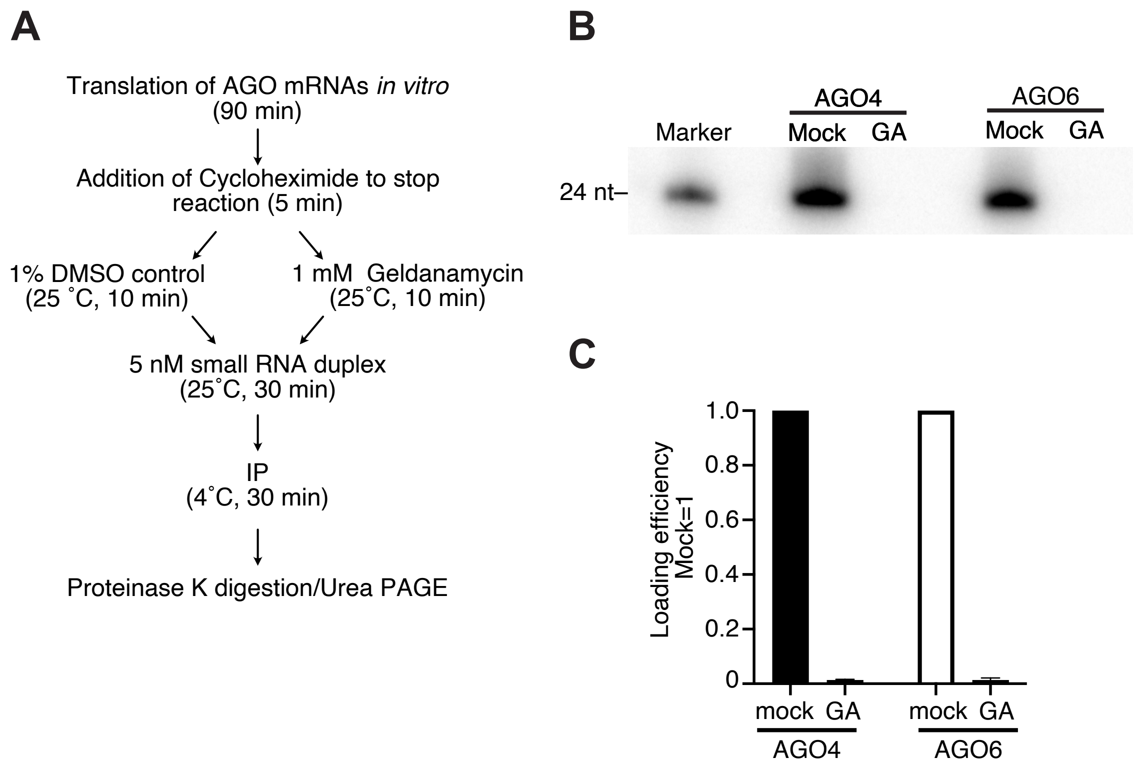

Supplementary Figure 2. Chaperon inhibitor abrogated loading of small RNAs by AGO4 and AGO6.

(A) Schematic for the RISC assembly with the chaperone inhibitor geldanamycin or DMSO control.

(B) RISC assembly in BY-2 lysate. HSP90 chaperone inhibitor geldanamycin abrogated loading of small RNAs in both AGO4 and AGO6, comparing to the mock group (DMSO control).

(C) Quantification of loaded siRNAs in (B). The band intensity of siRNAs was normalized to the value of mock group. The graphs show the mean ± SD from three technically independent experiments. See also Supplementary Table 3.

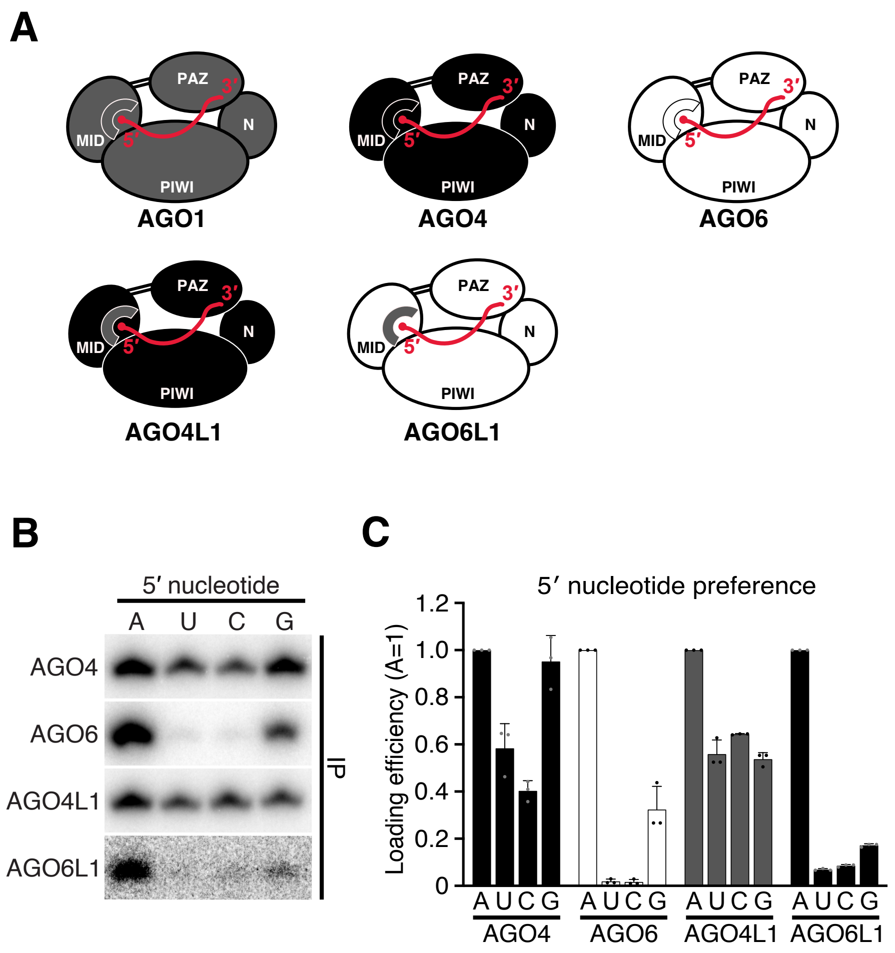

Supplementary Figure 3. Replacing the nucleotide specificity loop with that of AGO1 results in a slight change in the 5′ nucleotide preferences of AGO4 and AGO6.

(A) Schematic of chimeric AGO4 and AGO6 proteins with AGO1's nucleotide specificity loop.

(B) *In vitro* RISC assembly with chimeric AGO4/6 proteins. AGO4L1 and AGO6L1 showed similar 5′-nucleotide preference as AGO4 and AGO6, respectively.

(C) Quantification of loaded siRNAs in (B). The band intensity of siRNAs was normalized to the value of 5′ A. The graphs show the mean ± SD from three technically independent experiments. See also Supplementary Table 3.

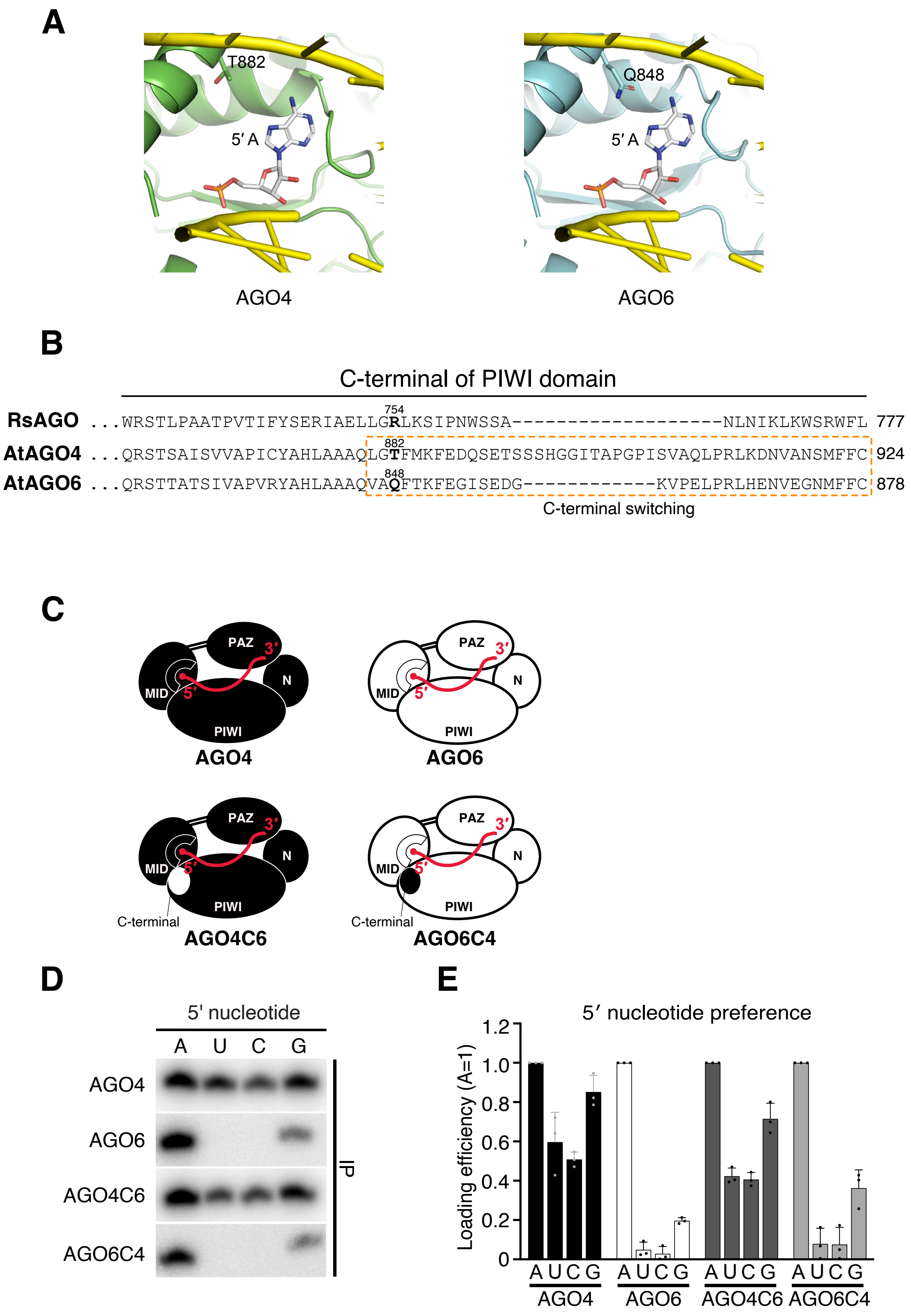

A
T882
5′ A
AGO4
Q848
5′ A
AGO6
B
C-terminal of PIWI domain
RsAGO ...WRSTLPAATPVTIFYSERIAELLGRLKSIPNWSSA-------------------NLNIKLKWSRWFL 777
754
AtAGO4 ...QRSTSAISVVAPICYAHLAAAQLGTFMKFEDQSETSSSHGGITAPGPISVAQLPRLKDNVANSMFFC 924
882
AtAGO6 ...QRSTTATSIVAPVRYAHLAAAQVAQFTKFEGISEDG------------KVPELPRLHENVEGNMFFC 878
848
C-terminal switching
C
PAZ
3′
N
MID
5′
PIWI
AGO4
AGO6
C-terminal
AGO4C6
AGO6C4
D
5' nucleotide
A U C G
AGO4
AGO6
AGO4C6
AGO6C4
IP
E
5′ nucleotide preference
Loading efficiency (A=1)
1.2
1.0
0.8
0.6
0.4
0.2
0
A U C G
AGO4
AGO6
AGO4C6
AGO6C4

Supplementary Figure 4. Swapping the C-terminal region of the PIWI domain between AGO4 and AGO6 results in a slight change in the 5′-nucleotide preference.

(A) Distance between the 5′ nucleotide of the guide strand and the C-terminal region of the AGO4’s (left) or AGO6's (right) PIWI domain in the structural model. Structural models of AGO4- and AGO6-RISC were created by superposing the structures of AGO4 and AGO6 predicted by AlphaFold2 with the recently solved structure of AGO10-RISC [PDB:7SWF] and then replacing the 5′ U of the guide strand with 5′ A *in silico*. In the structural models, Q848 in AGO6 is closer to the 5' A of the guide strand than T882 in AGO4.

(B) Sequence alignments of the C-terminal of PIWI domains of *Rhodobacter sphaeroides* Argonaute (RsAGO) and *Arabidopsis* AGO4 and AGO6. T882 in AGO4 and Q848 in AGO6 correspond to R754 in RsAGO. Sequences in the orange frame were switched between AGO4 and AGO6.

(C) Schematic of chimeric AGO4 and AGO6 proteins with each other’s C-terminal region of PIWI domain.

(D) *In vitro* RISC assembly with chimeric AGO4/6 proteins. AGO4C6 and AGO6C4 showed similar 5′-nucleotide preference as AGO4 and AGO6, respectively.

(E) Quantification of loaded siRNAs in (B). The band intensity of siRNAs was normalized to the value of 5′ A. The graphs show the mean ± SD from three technically independent experiments. See also Supplementary Table 3.
