# Supplementary material for: The mechanisms of siRNA selection by plant Argonaute proteins triggering DNA methylation
Source: Nucleic Acids Res. 2022 Dec 7;50(22):12997–3010. doi: 10.1093/nar/gkac1135 (PMC9825178; doi:10.1093/nar/gkac1135)
Supplement: gkac1135_Supplemental_Files [file gkac1135_supplemental_files.zip › Supplementary Table legends_revised.pdf]

Supplementary Table 1: DNA primers used in this study.

Supplementary Table 2: RNA oligos used in this study.

Supplementary Table 3: The quantitative data and p-values in Figure 1–5 and Supplementary Figure 1–4.
